# Extrinsic factors influencing gut microbes, the immediate consequences and restoring eubiosis

**DOI:** 10.1186/s13568-020-01066-8

**Published:** 2020-07-25

**Authors:** Ousman Bajinka, Yurong Tan, Khalid A. Abdelhalim, Güven Özdemir, Xiangjie Qiu

**Affiliations:** 1 Respiratory Department, Xiangya Hospital, Central South University, Changsha, 410008 Hunan People’s Republic of China; 2 Department of Medical Microbiology, Central South University, Changsha, 410078 Hunan China; 3 China-Africa Research Center of Infectious Diseases, School of Basic Medical Sciences, Central South University, Changsha, 410078 Hunan China; 4 Section of Basic and Industrial Microbiology, Department of Biology, Faculty of Science, Ege University, Izmir, Turkey

**Keywords:** Gut microbiota, Dysbiosis, Mediterranean diet, Inflammatory bowel diseases

## Abstract

From the emerging studies, the more diverse the microbial population in the gut, the healthier the gut. Health benefits are associated with the functional characteristics of these diverse microbial genes. Extrinsic factors causing dysbiosis are extensively studied however, linking the varying degree of consequences to the respective factors and therapeutic possibilities are not explored at length. This review aims to examine from previous studies and put forward the types of dysbiosis, the immediate consequences and the scientific approaches to restore disrupted microbiota. Dietary supplements are found to be one of the factors contributing profoundly to the alteration of gut microbiota. While diet rich in fibre and fermented food established a diverse microbiome and produce vital metabolites, high fat, animal proteins and high caloric carbohydrate are as well relative to dysbiosis among infants, adult or diseases individuals. The intermittent fasting, feeding methods, the pH and water quality are among the factors associated with dysbiosis. Prebiotics and Probiotics maintain and restore gut homeostasis. Antibiotic-induced dysbiosis are relatively on the spectrum of activity, the pharmacokinetics properties, the dose taken during the treatment route of administration and the duration of drug therapy. The higher the altitude, the lesser the diversity. Extreme temperatures as well are related to reduced microbial activity and metabolism. Delivery through caserium-section deprived the newborn from restoring valuable vaginal bacterial species and the baby will instead assumed intestinal microbiota-like. While exercise and oxidative stress contribute even though moderately, fecal microbial transfer (FMT) also influence gut microbiota.

## Introduction

One of the recent human health and diseases are the factors associated with dysbiosis. Dysbiosis is the reduced diversity of gut microbiota and restoring the lost microbiota to a much diverse composition is eubiosis. Are dysbiosis a result or causation is still in the mystery, partly due to circumstantial or speculative nature of the gut mechanisms. These factors influencing gut microbiota are in a constant flow and the gut with repertoire of microbes is resilient enough to adjust to any variation. In addition to natural variations, the immune system, the intestinal mucosa, microbiota itself, diet and ingested drugs are major factors influencing dysbiosis. What lies illusive until today are the mechanisms underpinning dysbiosis. These mechanisms are not thoroughly explored due to our limited characterizing techniques. The intricate interactions among species such as, the role of parasites, virus, bacteriophages and fungi in addition to natural variations such as, stress, secretions of bacterial toxins etc. when explored, will add weight to the current existing knowledge. Until the science regarding the above pathological effects is bridged to the effects they do cause to dysbiosis, the role of microbiota in health and disease would not be better understood (Travisano and Velicer 2004; Sartor and Mazmanian 2012; Ma et al. 2019). Furthermore, some elements surface; due to the available biochemical niches in the gut, microbes developed resiliency towards the lifestyles of the host. For example, the time and type of food consume by the host are naturally checked. Each for dietary type is specific to group of distinct phyla or class of bacteria. The more abundant they are with respect to phyla, the more adaptable they are to any biochemical changes (Sartor and Mazmanian 2012).

Human metabolism is indeed well explored, energy is generally derived via fermentation and sulphate reduction of dietary and host carbohydrates. Our gut is a complex organ harboring trillions of microorganisms that reside both as commensal and pathogens. In a balance healthy gut, the pathogenic group are dominated by the commensals. These commensals balance the gut microbiota and hence no infection or significant shift towards pathogenic bacterial overgrowth is observed (Thaiss et al. 2016). Depending on the biochemical and physiological properties in human body, microbes are dispersedly distributed. They can be found in the mouth, on the skin, genital tract and in the gut. Acidophilic species such as *Streptococcus*, *Lactobacillus*, *Helicobacter Pylori*, *Candida* and *Peptostreptococcus* are found in the stomach, *Lactobacillus* and *Streptococcus* are found in duodenum, jejunum and proximal ileum (Travisano and Velicer 2004). The further you go from esophagus to the rectum, the less acidic it become and the number of bacteria are on ascending trend towards the colon. More than 100 species from *Actinomycinae*, *Bacteroidetes*, *Streptococcus*, *Clostridium* and *Corynebacteria* spp. are concentrated in the distal ileum. More than 1000 species mainly, *Bacteroidetes*, *Clostridium* Type IV and XIV and *Enterobacteriaceae* are found in the colon (Sartor and Mazmanian 2012; Thaiss et al. 2016). With the limited molecular and genomics techniques, the study of gut microbiome in health and disease is not species specific instead clusters of bacteria forming genera. Studies have found that *Firmicutes* and *Bacteroidetes* with 64% and 23% respectively are the most common in individuals. Nevertheless, *Actinobacteria* and *Proteobacteria* are also present in varying percentages (Sartor and Mazmanian 2012). Until today, there is no such as to what is a normal gut microbiota. However, abundance in *Proteobacteria* in the gut is studied to be associated with inflammation at the mucosal wall and this, in effect leads to disruption of epithelial lining. Excess *Firmicutes* and metabolic disorders are associated with obesity (Kasai et al. 2015; Ley et al. 2008).

The knowledge gap in gut microbiota medicinal approach is not bridged completely. Microbes residing in the intestinal tract influence local and systemic process of the host such as supplying vitamin, maturation of mucosal immunity, nutrient transformation, influence on the brain and neurotransmitter (Peng et al. 2018). Balance microbiota is studied to be a more diverse population, which is profoundly associated with a healthy gut. Based on the recent Metagenomics analysis, a healthy human gut comprises mainly *Bacteroidetes*, *Firmicutes*, *Actinobacteria* and *Proteobacteria* (Arumugam et al. 2011). Any shift in these communities of gut bacteria results in an imbalance or dysbiosis in the gut microbiota. Dysbiotic or less diversed microbial community in the gut is studied to be associated with the onset of diseases or the driving force behind diseases mechanism such as metabolites (Kasai et al. 2015). The longevity of a host is determined by the ability of specific microbes contributing to the range of essential functions that ascertain the normal healthy function or restoring a balance diversity (eubiosis) in the gut. An essential function in the gut herein refers to the digestive role, absorption of nutrients and excretion of waste (Travisano and Velicer 2004). Gut microbiota stimulates the immune system and plays a key role in cell regeneration, produce enzymes, vitamin K and biotin. They enable the digestion of polysaccharides and influence bacteria in the gastrointestinal tract (GI) tract and influence host’s metabolism. Polysaccharides, as prebiotics components, are being speculated to confer positive effects in managing metabolic diseases like obesity and diabetes (Lupp et al. 2007).

**Fig. 1:**
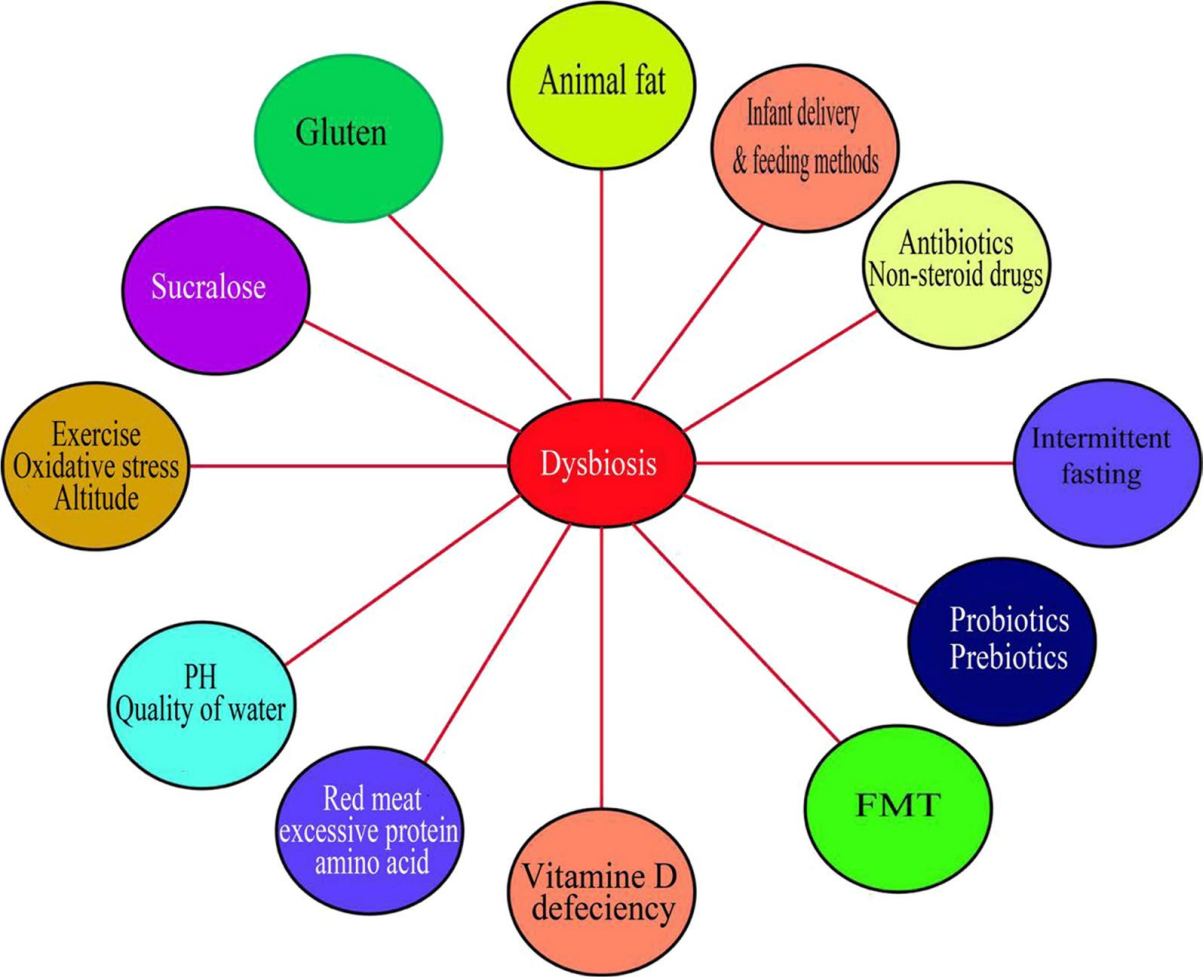
Factors causing alteration to gut microbiota. Diet rich in protein, animal fats and high carbohydrate, sucralose and diet containing gluten all contributed to dysbiosis. Exercise and intermittent fasting are studied to both starve the bad microbes and clean the gut. Method of delivery for the newborn baby and the feeding methods determine the childhood immunity and this period is crucial for the development of human life. The pH level or water quality are as well among the factors associated with dysbiosis. In addition, drugs including antibiotics, non-steroids anti-inflammatory drugs, Prebiotics and Probiotics adversely affected the gut microbiota composition. Other factors are vitamin D deficiency, oxidative stress, temperature and fecal microbial transfer

Apart from heredity factors that are even known to have lesser influence on the gut, drugs such as antibiotics, non-steroid drugs, chemotherapy, radiotherapy and poor nutrition are the extrinsic factors. Diseases, physical and psychological stress, are the intrinsic factors studied to cause dysbiosis. The resulting consequences of all these factors are dysregulation of immune system, leading to autoimmune diseases, onset of disease such as inflammatory bowel diseases (IBD), colorectal cancer and metabolic disorders such as diabetes. Number of studies have established facts on the complex microbial community in the gastrointestinal tract as essential for human health (Ley et al. 2008; Frank et al. 2007). While there are other factors inflicting alteration in the gut microbiome, causing dysbiosis and leading to number of complications, this review is set to examine the extrinsic factors influencing gut microbes, the immediate consequences caused and the mechanisms studied to reinstate the gut to a healthy function. Since it is a newly born discipline, we draw much attention on the number of publications centered on dysbiosis and extended the scope dating back to 20 years (Fig. 1) (Additional file 1: Figure 1).

## The effects of dietary on gut microbiota

Apparently, we are what we eat, so we should eat the right food. Food can be served as medicine, preventing and treating diseases and in fact food was used as antimicrobial in the era of pre-antibiotics. Our dietary patterns have direct influence on the occurrence and variety of bacteria in the gut, which consequently affects our health. Fascinating findings on food science do not only confer high nutritive benefits to humans, but is also the medicinal use attached to food industries. It is believed to be a major breakthrough in medicine; simply changing diet can save us from the cost of treatment. Modern diets or western diets caused lots of preventable diseases like asthma, obesity, multiple sclerosis, etc.

The effects and influences of diets on our gut microbiome is not a new area of study. The meta-transcriptomic studies have revealed profound evidence and set aside multiple conditions. The ideal microbiota is achieved through the capacity of the microbial members to metabolize sugars, and the ‘reflective adaptation’ of the microbiota to the available nutrient in the intestine (Zoetendal et al. 2012). Foods are to be explored and going far and wide means eating more and wild, thus the diversity of microbes, and a healthy gut microbiota. The transient changes brought to the gut microbiota composition by food are mainly due to fish, meat and fibers with durable effects. It was the classes of food that was put into the scope of nutrition studies. However, with gut microbiota medicine, the role of gut microbiota in diseases is attracting much attentions. One diet can contain more than two macronutrients and effects changes on gut microbiota while significantly influencing the metabolic output (Qiu et al. 2020).

## Dietary fibre

Studies have correlating results on the beneficial effects of dietary fibreon human metabolism. Meta-analysis showed a clear link between dietary fibre and wide range of pathologies (Qiu et al. 2020). One of the recent interventions showed that dietary fibre could significantly reduce the insulin resistance among patients with type 2 diabetes. Moreover, clear links have been seen in dietary fibreshifting the microbiota and beneficial metabolites such as butyrate (Silva et al. 2020). While feeding on all the classes of foods is imperative to our health, diet rich in fibre stands prominent in maintaining the diversity of the gut microbiota (Zhang et al. 2013). For example, colonic microbiota is determined to a larger extent by the availability of microbiota-accessible carbohydrates (MACs) found in the dietary fibre we consume. Furthermore, the more extreme we go with strict ‘animal-based’ diets or ‘plant-based’ results into some extent of dysbiosis (David et al. 2013). Fibre has been the most studied food. Its influence on the small intestinal bacteria is understood to be enriching the gut with variety of benign microbial metabolism.

A crossover or a comparative study based on diets showed that, the influence of diets highly rich in resistance starch or non-starch polysaccharide fibre resulted in the enrichment of diverging pattern in bacterial growth (Gibson 2017). In fact, during the microbial digestion or breakdown of food residue or chyme, finest food particles or molecules cross the epithelial barrier to the blood stream or serum. If these molecules are highly concentrated with fibre, they serve as an antioxidants and hence cleansing mechanism in the blood stream. In contrast, uremic toxins are secreted into the gut with an increased protein fermentation as well as phenols, indoles, thiols and ammonia (Schultz et al. 2009). These are either anti-inflammatory or antioxidant in the gut. Microbial fermentation existing with the uptake of dietary fibre is by far the maximum utilization of energy in the gut. The indigestible diet rich in fibre reached the colon when the host cells could not secrete specific enzyme to act on it, thus they are fermented on by the gut microbes (Silva et al. 2020). The increase in abundance of *Firmicutes* over *Bacteroidetes* enable the gut to provide more enzymes for digesting any indigestible polysaccharide supplied from dietary fibre (Qiu et al. 2020). The more the microbes digest these polysaccharides, the more monosaccharides and hence an increased production of short chain fatty acid (SCFA) by the intestinal mucosa. Monocarbides are studied to augment lipid fat stores in peripheral tissue through the de novo hepatic lipogenesis with transcribed genes (Spanogiannopoulos et al. 2016). Monocarbides produce SCFAs that are specific for various energy supply such as colon cell proliferation and differentiation by Butyrate. Supply of energy lipogenesis in peripheral tissues is modulated by acetate and gluconeogenesis in the liver by propionate (Guarner and Malagelada 2003; Bäckhed et al. 2004).

## High fat diet

High fat diet intake is found to be have detrimental effects on the penetrability of the mucus layer and also obstruct metabolic functions. These studies were conducted on a separate timeline and yet, correlating results where achieved. An increased in *Firmicutes* and decrease in relative abundance of *Bacteroidetes* is studied to be associated with high fat diet (Zhang et al. 2012). *Akkermansia muciniphila*, *Actinobacteria* and lactic acid bacteria with their protective functional genes influence the gut microbiota through diet rich in unsaturated fat. In contrast to unsaturated fat, saturated fat in mice model, lipopolysaccharide (LPS) production is prominent. Also the expressions of TLR4 and TLR2 have increased significantly (Yee et al. 2013). A very strong antimicrobial activity such as low pH is produced in the gut through the re-absorption of bile acids in the distal ileum. These bile acids are emulsified through the high intake of dietary lipids. High fatty acid diet is associated with ulcerative colitis (UC) and increase risk of colon cancer. The dietary administration of insulin is believed to prevent the advent of these detrimental effects (Frank et al. 2007). Furthermore, butyrate is playing a role in preventing oxygen induced gut microbiota dysbiosis and due to this, the consumption of dietary fibre is medically recommended for maintaining the normal mucosal barrier function in the gut (Zoetendal et al. 2012). In addition, Butyrate reduces bacterial translocation, improves the organization of tight junctions and stimulates the synthesis of mucin, a glycoprotein maintaining the integrity of the intestinal epithelium (Travisano and Velicer 2004).

## Animal fats and amino acids

Diet based on animal fats and amino acids show an increased rate of *Bacteroides* and low protein while diet much depending on carbohydrate gives abundance in phylum *Prevotella* (Saha et al. 2017). Based on rat model, instead of *Bacteroides*, high-protein diet is associated with a decreased abundance of *Clostridium species* and *Faecalibacterium prausnitzii* (Liu et al. 2014). The production of branch chain fatty acids is prominent however, prominent as well is a potential toxic substance such as ammonia, sulfide and *N*-nitroso compounds, which are associated with high-protein diet (Wang et al. 2013). Excess in protein and amino acids synthesis more nitric oxide that influence the gut microbiota, leading to IBD, such as Crohn’s disease (CD) (Magee et al. 2000).

## Sucralose

An increased in the gut bacterial pro-inflammatory genes and unpredictable faecal metabolites was observed in mice given sucralose for 6 months (Zhang et al. 2012). Another interesting study reveals that sucralose, aspartame and saccharin can cause dysbiosis in gut microbiota. When compared to those fed with non-sucralose, rats were seen with significantly higher proportions of *Clostridia*, *Bacteroides*, and total aerobic bacteria in their gut and a rise in gut pH (Schultz et al. 2009).

## Mediterranean diet

Mediterranean diet is one such diet enhancing a healthy gut. This diet is characterized by olive oil and oily fish (rich in mono and polyunsaturated fatty acids). Also is a high intake of fruit and vegetables (rich in fibre, antioxidants and vitamins), and whole grains and nuts (Sartor and Mazmanian 2012). To attain the most efficient nutrients from this type of diet, it has to be daily or weekly consumption based on the standardized food pyramid. One of the foods still in the debate between regulatory agencies and gut microbiologist is the high-intensity sweeteners used as sugar alternatives. These are believed to be minimal calories. They are regarded healthy to consume however, recent studies have shown that these substitutes may have some negative effects on the gut microbiota (Nettleton et al. 2016).

## Vegan’s diet

The debate on vegans eating healthy food better than non-vegans is still strong and to extent a sensitive topic. Considering the bad experiences with food, our apetite can be influenced and this affect the next food to choose. Advertisement, culture or people around can influence what meal we have on the table. Several studies including the one that recruited 15 vegans and 16 omnivores revealed amazing results. Despite, the differences in the serum metabolites between both groups, the gut bacterial community shows insignificant diversity (Wu et al. 2016). This must not be a good news for both the vegans and omnivores group. Furthermore, another randomized trial on 10 human omnivores were on either a high fat and low fibre diet or low fat and high fibre for a period of 10 days corroborated the results of this finding. Again, the microbiome composition has not been shaken and the differences in short chain fatty acid production was not significant. Perhaps the experiments should be extended for a longer period of time. This may assure bacterial derived metabolome to be influenced by being a vegan or not (Wu et al. 2016).

## Gluten

Based on the studies of micro nutrients, gluten-free bread reduces the microbiota dysbiosis. Meaning they created a well-balanced microbial community as seen in vitro animal study (Bonder et al. 2016). People with gluten sensitivity or coeliac disease must have welcomed this news after this study was published. 21 healthy people with a profound diversity in the gut microbiota, just after 4 weeks on a gluten-free diet showed a balance diversity. However, among these, are good number who showed a lower abundance of the most common and important microbial species in the gut (Lebwohl et al. 2017). It may be too soon to conclude anything as scientists are still in the lab. In contrast to the coeliac disease related research above, until today, some people avoid gluten and yet they do not have the coeliac diseases or even proven gluten intolerance. In fact, from one of the observational studies, heart diseases were seen with an increasing rate among gluten avoiders (Schroeder et al. 2018). Moreover, it is speculated that perhaps the reduced consumption of whole grains is not good for maintaining the health of some organs. However, more knowledge is required to shed more light on these assumptions.

## Dietary gut therapy

With the overfeeding hypothesis, homeostatic are disrupted due to over feeding, leading to dysbiosis, metabolic disorders and diseases (Lachnit et al. 2019; Kim et al. 2014). This is eminent in diet with low-fibre content, easily digestible and energy-dense, all influence the functional genes. Intermittent fasting is ascertained to confer changes in cellular pathways, preventing the development of diseases such as rheumatoid arthritis, obesity, hypertension and asthma, and delay in aging (Longo and Mattson 2014). Human has a set of factors that form mucosal environment and any uncontrolled growth of bacterial population through overfeeding with western-style diet (WSD) impairs carbon to nitrogen to phosphorous ration. Since these diets are easily digestible by both human cells and the microbes, diets rich in sugars, fats, and proteins with small amounts of fiber, lead to imbalance nutrient in the gut. This result to depilation of mucosal barrier and the onset of infections. However, the release of bacterial by-products stimulate the functionalities of immune cells through non-self-recognition of immune reactions thereby initiating immune response (Sakaguchi et al. 2020; Lachnit et al. 2019). This area of research is an inexhaustible discipline and until today, not much has been done especially when it comes to the bad experiences with food. Much often, many people are still struggling with the side effects of popular restrictive diets on gut health. The strict vegans would attest to this and those feeding on raw food or “clean eating” diets, diets free of gluten. Also with side effects are those on FODMAP (fermentable oligosaccharides, disaccharides, monosaccharide, and polyols), irritable bowel syndrome (IBS) treatment alternative diets (Lebwohl et al. 2017).

## Antibiotics and drugs

The effects of antibiotics can be attributed to the mode of mechanism, the class of antibiotic, the degree of resistance of the antibiotic used and the dosage used during the treatment. In addition, the route of administration, the pharmacokinetic and pharmacodynamics properties and the spectrum (broad or narrow) all relatively alters gut microbiota composition (Rogers and Aronoff 2016). Non-steroidal anti-inflammatory drugs are commonly used daily however, these are causative agent for stomach ulcers. Among the consequences, metabolic disorders are extensively studied (Freedberg et al. 2015). Microbes themselves are studied to regulate the drug mechanisms. To have a profound insight as to gut dysbiosis, in addition to the type of drugs used, the existing microbiota, immune system and the intestinal mucosa are the prerequisites. While it is understood that only the bacterial communities are explored in the quest to establishing the role of gut in health, other gut microbial species are somehow contributing to the dysbiosis status of the gut microbiota. Until today, long term natural fluctuations such as diseases outbreaks and mutations of the strain might as well rendering alteration to the gut even before antibiotic treatments. Proton pump inhibitors (PPIs), antidepressants, metformin, laxatives and oral steroids are all studied to confer profound dysbiotic effects on the gut.

## Prebiotics

Among the well characterized bioactive compounds are phytochemicals and Prebiotics, polyunsaturated fatty acids (PUFAs). Prebiotics have proven to be source of fermentation products such as SCFAs that confer diverse biological benefits to the gut (Hill et al. 2014). While PUFA effects advantage to the host immunity and metabolism, phytochemicals are effective bioactive for anti-inflammatory effects, antioxidants, immune modulatory, anti-carcinogenic and anti-estrogenic effects. In addition to these effects, phytochemicals are studied to exert Prebiotics-like effects on gut, thereby inhibiting pathogenic bacteria. In doing so, they are stimulating the growth of the benign bacteria (Laparra and Sanz 2010).

## Oxidative stress

A good number of studies have shown a correlation between increased oxidative stress and reduced gut microbial diversity. The modern dietary habit or the so called western-style diet is rich in fat and refined sugars. These in large quantities, cause an increase of the inflammatory status with reactive oxygen species (ROS) production. Subsequent ROS stimulate the inflammatory cascade (Bellavia et al. 2013). The stress induced by ROS production defined as oxidative stress. Oxidative stress consist of biological system for the process of detoxification and secondary damage repair (Liu et al. 2018). The new study though not supported with enough literature is the potential role of heat shock proteins (HSPs) in the pathogenesis of IBD. HSPs are indentified to play a role in folding, translocation and degradation of intracellular proteins under normal and stressful conditions. They can stimulate an immune response, both innate and adaptive and hence primary targets of the autoimmune response. They are conserved molecules with similar sequences in bacterial and human orthologs (molecular mimicry) (Zhang et al. 2013).

## The scoioeconomic status

The variation in diversity and composition of gut microbes is largely determined by the economic status of the country or region the baby is born and a child is raised. This is due to the available food choices left as the options for mothers to feed their babies. It is a fundamental scientific principle that malnourished individuals are prone to unsteady health conditions and this implies to undernourished infants. Infants that are undernourished lead to dysbiosis condition and abundance of enteropathogens such as *Enterobacteriacea* being prominent in their gut (Reijnders et al. 2016). A comparative analysis done on two separate studies, that involves infants from rural Africa and European children showed pattern that are directly correlating with the type of diet they consume. Diet dominated by starch, plant polysaccharides and fibre enable the abundance in *Bacteroides* phyla at 57% and *Actinobacteria* at 10% (Lee et al. 2017). For the European children, due to their diet rich in sugar, animal protein and starch, the abundance of the bacterial population showed a reduce diversity (Alexander et al. 2017). Furthermore, Pretovella, which is a major SCFA were found exclusive to the rural African children microbiota and the same trend is persisting even in healthy human adult consuming high amounts of carbohydrates and some forms of simple sugars (O’Keefe et al. 2015). SCFAs are found to be very significant to the health of gut microbiome and this is manifested in its role in anti-inflammatory mechanisms (Spanogiannopoulos et al. 2016). Furthermore, SCFAs (acetate, butyrate and propionate) are studied to be signaling molecules that help in the maintenance of the integrity of colonic epithelium, lipid metabolism, appetite regulation and glucose homeostasis (Morrison et al. 2016).

However, a decreased condition is found among individual feeding on low MACs, the part of dietary fibre that bacteria in large intestine can feed on (Spanogiannopoulos et al. 2016). Unfortunately, nowadays the abundance of MACs is observed in a decreasing trend in the western diet. The establishment of MACs as a major factor for restoring the gut diversity. Mice supplied with a diet of low MAC results to a massive reduction in their gut microbial diversity (Deehan et al. 2017). There is hope for children affected with under nutrition since the study using gnotobiotic mice ascertained that some microbial species have the power to restore growth impairments transmitted by microbiota isolated from malnourished children (Martínez et al. 2010). Varying causative factors to dysbiosis in the gut microbiota with consequences of which some have alternative therapeutic approaches (Table 1).

**Table 1:** Factors, types of dysbiosis caused in the gut, consequences and alternative therapeutic approaches

Extrinsic Factors	Observations in the gut	Alteration in the gut	Alternative remedies
Dietary			
High-fat/high-sugar	^a^Adherent-invasive *Escherichia coli* (AIEC)	Permeability in the intestinal mucosal and reduced expression of tight junction proteins zo-1 and occluding. Compromising selective absorption and triggers the onset of metabolic endotoxemia (Martinez-Medina et al. 2014)	PUFA and conjugated linoleic acid (CLA), phytochemicals, Mediterranean diet
A high-protein diet	^a^Potentially toxic substances such as sulfide, indole, ammonia and *N*-nitroso compounds)	Deleterious and toxic for the intestine (Martínez et al. 2010)	PUFA and conjugated linoleic acid (CLA), phytochemicals, Mediterranean diet
Excess dietary intake of protein and amino acids	^a^Synthesis of nitric oxide (NO)	Development of an obesity-associated microbiota, ulcerative colitis (Martinez-Medina et al. 2014), lead to DNA damage (Martinez-Medina et al. 2014; Irrazábal et al. 2014)	PUFA and conjugated linoleic acid (CLA) due to anti-inflammatory characteristic
Excess nutrient intake	^a^Firmicutes	Obesity related to inflammatory metabolic disorders culminating from dysbiosis (Frank et al. 2007)	Probiotic (*Lactobacillus paracasei*) ^a^circulating levels of ANGPTL4 and reduces body fat
Vitamin D deficiency	Induced dysbiosis in the gut microbiota	Play a role in the pathogenesis of IBD and ^a^risk of colitis (Ananthakrishnan 2015)	Vitamin D supplementation had an anti-inflammation due to the inhibition of pro inflammatory genes such as TNF genes
Red meat (thiol-containing amino acids)	^a^Sulfate-reducing bacteria (e.g. *Desulfovibrio* spp., in the intestine	^b^Mucus formation, inhibits methylation of DNA and ^a^ the generation of reactive oxygen species (Ananthakrishnan 2015)	Stress such as exercise under sun or heat exposure) is recommended
Saturated fatty acids and low in polyunsaturated fatty acids (vegetable oil) mouse model	^a^*Bilophilawadsworthia*	Inflammatory reaction mediated by Th1 cells and colitis, high bad low-density lipoprotein (LDL) cholesterol level (Ananthakrishnan 2015; Liu et al. 2020)	Feeding on mono-saturated fats and polyunsaturated fatty acids (vegetable oil)
Sucralose (aspartame and saccharin) in mouse model	^a^*Clostridia, Bacteroides* and total aerobic bacteria, rise in pH (Schultz et al. 2009)	Pro-inflammatory genes and unpredictable faecal metabolites (Zhang et al. 2012)	FMT, PUFA and conjugated linoleic acid (CLA), mediterranean diet
Gluten	Dysbiosis in microbiota, ^a^pathogenic bacteria	^a^ Risk of coeliac diseases or even proven gluten intolerance, constipation and diarrhea (Schroeder et al. 2018)	Gluten-free diet and hydration
Fasting and hibernation	^a^*Bacteroidetes* and *Verrucomicrobia* ^b^*Firmicutes* (more polysaccharides)	Capable of degrading mucin, suppress the immune system thus ^a^*Firmicutes* (more polysaccharides) the tolerance of the host to its microbes (Secor and Carey 2016)	PUFA and conjugated linoleic acid (CLA), phytochemicals, Mediterranean diet and hydrating
Starvation	^a^*Proteobacteria* in the gut	Risk of inflammation at the mucosal layers that leads to the breaking down of epithelial barrier called ‘leaking gut’ (Couteau et al. 2001)	PUFA and conjugated linoleic acid (CLA), phytochemicals, Mediterranean diet
Antibiotic (dextran sulfate sodium)	^a^Liberation of sialic acid 2, 3-linked sialylatedglycans from the intestine (mouse model), Huang et al. 2015) ^a^*Salmonellaenterica* serovar *Typhimurium* and *C. dificile* (Ng et al. 2013)	Intestinal inflammation, IBD and persisting diarrhoea	Butyrate is anti-inflammatory effect (expressed by reducing the production of pro-inflammatory factors such as Nf-kb) making it a valuable ally in the treatment of IBD symptoms (Longo and Mattson 2014)
Non-steroidal anti-inflammatory drugs			
Metformin (hepatic gluconeogenesis inhibitor)	^a^ in *E. coli*	Although used as the standard medication for type 2 diabetes, it cause gut microbiota dysbiosis(Chassaing et al. 2015)	Probiotics (*Lactobacillus rhamnosus*)
Proton-pump inhibitors	^a^*C. difficile*	*C. difficile* associated diarrhoea (Freedberg et al. 2015) and hepatic encephalopathy in cirrhotic patients (Tralongo et al. 2017)	Probiotics (*L. rhamnosus*)ensure eubiosis (Tralongo et al. 2017), Fecalmicrobial transplantation (Garza and Dutilh 2015)
Aspirin, ibuprofen and naproxen (a month long intake)	^a^*Bacteroidaceae* and *Enterobacteriaceae* (Rogers and Aronoff 2016)	Risk of stomach ulcers	Proton-pump inhibitors are often prescribed in combination
Emulsifiers (polysorbate-80 and arboxymethylcellulose) mice	^a^*Proteobacteria* in the mucus was enriched, ^b^*Verrucomicrobia* and *Bacteriodetes*	Low grade inflammation, ^a^ risk if obesity and metabolic disorders (Chassaing et al. 2015)	While avoiding processed foods as much possible, avoid eating late at night
Artificial cleansing for colonoscopy	Microbial load in the gut is ^b^by about 30-fold	^b^Bacterial diversity in the short term but restored after approximately 14 days (Parnell and Reimer 2012)	Prebiotics (inulin fibre) gives SCFAs including butyric acid
Prebiotics (oligofructose)	^a^*Bacteroidetes*^b^*Firmicutes* in ob/ob mice and in rats	Genetically prone to develop obesity and insulin resistance (Parnell and Reimer 2012)	*A. muciniphila* thickens the mucus layer, thereby ^b^ gut permeability, endotoxemia, obesity and preventing inflammation
Probiotics (*A. muciniphila*)	Mucin degrading bacteria	^a^Severity in colitis models (Kang et al. 2013)	Administer only when relevant
Other factors			
Oxidative stress	^a^*Enterobacteriaceae*	Secretion of lipopolysaccharide (LPS) intensifies response to inflammation (Lupp et al. 2007)	PUFA and conjugated linoleic acid (CLA), phytochemicals, Mediterranean diet
Temperature of 6 °C (mouse model)	^a^*Firmicutes* and ^b^*Bacteroidetes*	Thus activating non thermopile groups of bacteria (Chevalier et al. 2015)	Maintaining normal body temperature is highly recommended
Mode of delivery (caesarian section)	^a^epithelial bacteria ^b^*Bifidobacteria* spp. *lactobacillus* spp.	High rate of asthma and allergies (Bäckhed et al. 2015)	Delivery through virginal canal and Probiotics supplementation of missing taxa
Feeding method (formula fed)	Profound influence on microbiota composition	Overweight and risk of childhood obesity (Bäckhed et al. 2015)	Breast feeding supply *Bifidobacterium longum* subspp*. infantis*, L*actobacillus*

^a^Denotes increased in abundance, expansion etc

^b^Denotes decreased in abundance, reduced etc

When we lose the diversity of our intestinal microbiota, we are prone to other factors that can be internal or external. Dwelling on these factors, at first, the restoration of these diversities needs to be checked. The administration of MACs and in combination of supplementing the missing taxa is studied to be very efficient (Hooper et al. 2003). In line with this intervention, a study that dealt with malnourished children shows correlating results; gnotobiotic mice revealed that, there are specific microbial species found in malnourished individuals that can cause growth impairments. The administration of some missing taxa are seen to have the ability to restore these impairments. If this study holds for similar results, then there is a big hope that microbes can be used to restore growth impairments from malnourished children and hence another new therapeutic intervention in countering the negative effects of under nutrition (Odenyo et al. 2001). Host intrinsic factors such as genetics can influence the microbial population. However it is not well pronounced when compared with the effects of diet and antibiotics on the microbiota. This was in agreement with results emerging from studies on individuals with contrasting geography and locations, twin studies, and people settling in rural and industrial settlements (Ayeni et al. 2018; Vangay et al. 2018).

Dietary interventions to mitigate and establish positive response of our gut microbes would definitely pave a great way as to determining the effects of microbes and our metabolic response. For example, fibre as is a key nutrient for a healthy microbial composition is hoped to remedy series of autoimmune disorders while the fat and sugar are yet to proof beyond any medicinal evidence. From the time of the edition of this review and even beyond, clinical trial based studies are underway and are not far reaching scientific grounds to prevent the human population against autoimmune and inflammatory diseases. The intervention of the Probiotics and Prebiotics and the FMT are also a competing factor to bring in a desirable change in the gut to ascertain a healthy and normal composition. FMT *proves C. difficile* infection cured more than 90% of cases (Garza and Dutilh 2015).

## Supplementary information

**Additional file 1: Figure 1.** Factors causing alteration to gut microbiota. Diet rich in protein, amimal fats and high carbohydrate, sucralose and diet containing gluten all contributed to dysbiosis. Exercise and intermittent fasting are studied to both starve the bad microbes and clean the gut. Method of delivery for the newborn baby and the feeding methods determine the childhood immunity and this period is crucial for the development of human life. The pH level or water quality are as well among the factors associated with dysbiosis. In addition, drugs including antibiotics, non-steroids anti-inflammatory drugs, Prebiotics and Probiotics adversely affected the gut microbiota composition. Other factors are vitamin D deficiency, oxidative stress, temperature and fecal microbial transfer.

## Data Availability

Not applicable.

## Abbreviations

GI: Gastrointestinal tract
IBD: Inflammatory bowel diseases
MACs: Microbiota-accessible carbohydrates
SCFAs: Short chain fatty acid
LPS: Lipopolysaccharide
UC: Ulcerative colitis
CD: Crohn’s disease
WSD: Western-style diet
FODMAP: Fermentable oligosaccharides, disaccharides, monosaccharide, and polyols
IBS: Irritable bowel syndrome
PPIs: Proton pump inhibitors
PUFAs: Polyunsaturated fatty acids
ROS: Reactive oxygen species
HSPs: Heat shock proteins
CLA: Conjugated linoleic acid
LDL: Low-density lipoprotein
