# Supplementary figures and images for: Extrinsic factors influencing gut microbes, the immediate consequences and restoring eubiosis
Source: AMB Express. 2020 Jul 25;10:130. doi: 10.1186/s13568-020-01066-8 (PMC7381537; doi:10.1186/s13568-020-01066-8)

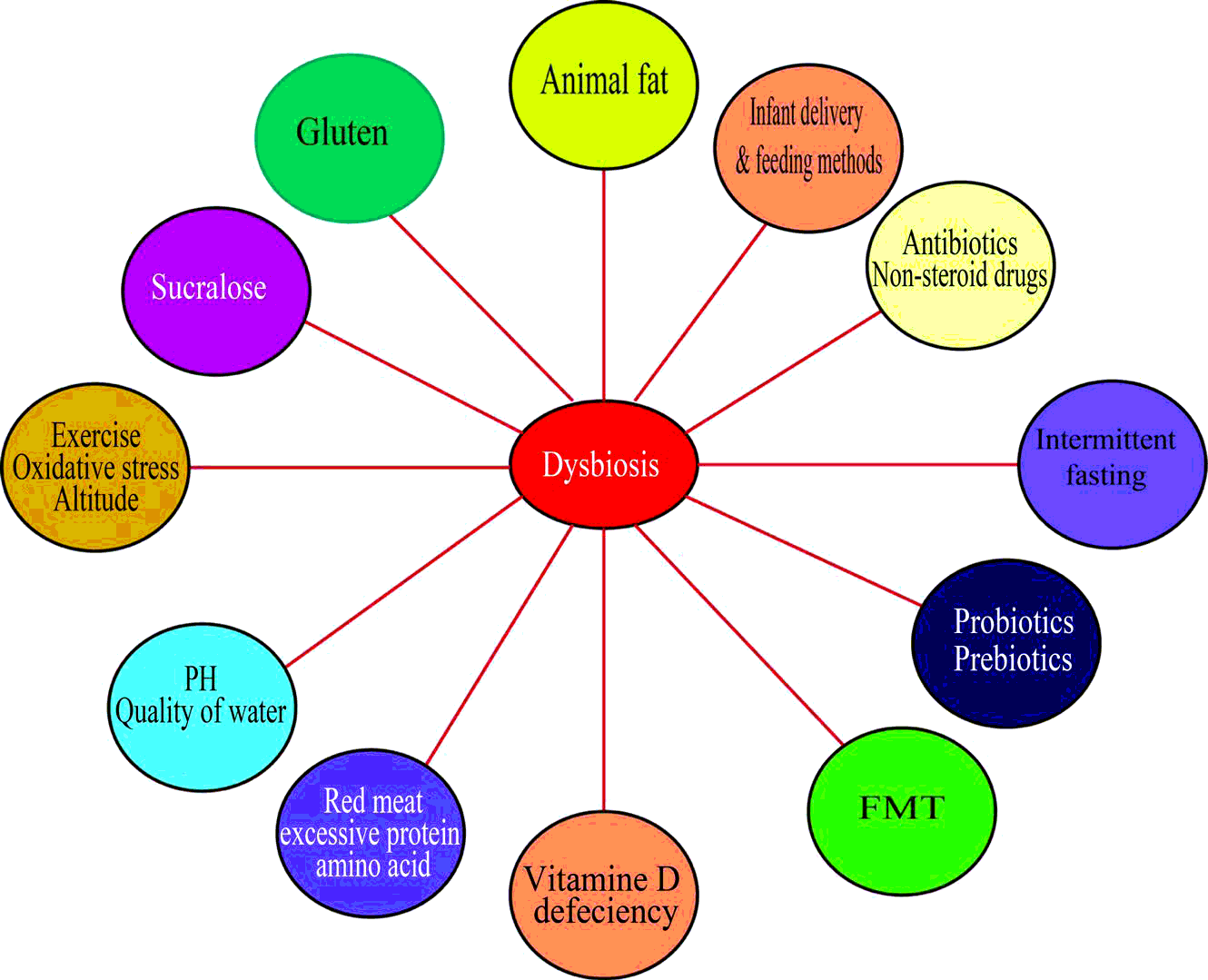

Supplement: Supplementary file 1 — Additional file 1: Figure 1. Factors causing alteration to gut microbiota. Diet rich in protein, amimal fats and high carbohydrate, sucralose and diet containing gluten all contributed to dysbiosis. Exercise and intermittent fasting are studied to both starve the bad microbes and clean the gut. Method of delivery for the newborn baby and the feeding methods determine the childhood immunity and this period is crucial for the development of human life. The pH level or water quality are as well among the factors associated with dysbiosis. In addition, drugs including antibiotics, non-steroids anti-inflammatory drugs, Prebiotics and Probiotics adversely affected the gut microbiota composition. Other factors are vitamin D deficiency, oxidative stress, temperature and fecal microbial transfer. [file 13568_2020_1066_MOESM1_ESM.png]
